# Supplementary material for: The Drosophila histone methyl-transferase SET1 coordinates multiple signaling pathways in regulating male germline stem cell maintenance and differentiation
Source: bioRxiv. 2024 Feb 14:2024.02.14.580277. Preprint. [Version 1] doi: 10.1101/2024.02.14.580277 (PMC10888844; doi:10.1101/2024.02.14.580277)
Supplement: Supplement 1 [file NIHPP2024.02.14.580277v1-supplement-1.pdf]

bioRxiv preprint doi: https://doi.org/10.1101/2024.02.14.580277; this version posted February 14, 2024. The copyright holder for this preprint (which was not certified by peer review) is the author/funder, who has granted bioRxiv a license to display the preprint in perpetuity. It is made available under aCC-BY 4.0 International license.

Supplemental Information

Materials and methods

Fly strains and husbandry: Flies were raised under standard yeast/molasses medium at 25℃ unless stated otherwise. The following flies were used: *nos-Gal4 (with VP16)/Cyo* (Van Doren et al., 1998), *nos-Gal4 (without VP16* or *ΔVP16)* on the 2nd chromosome (from Yukiko Yamashita, University of Michigan, USA), *UAS-set1 RNAi* (Bloomington *Drosophila* Stock Center, BL33704), *UAS-mCherry RNAi* (Bloomington *Drosophila* Stock Center, BL35785), *tj-Gal4/Cyo* (Tanentzapf et al., 2007) , *bam-Gal4/TM6B* (Chen and McKearin, 2003), *bam-Gal80* on the third chromosome (from Juliette Mathieu and Jean-René Huynh, Collège de France, France), *UAS-GFP.nls* (Bloomington *Drosophila* Stock Center, BL4775), *UASp-FRT-EGFP-set1 (WT)-PolyA-FRT* (This study), *UASp-FRT-EGFP-set1 (WT rescue)-PolyA-FRT* (This study), *UASp-FRT-EGFP-set1 (Mutant rescue)-PolyA-FRT* (This study), mad^{12}, *FRT40A/Cyo* (Bloomington *Drosophila* Stock Center, BL58785), $stat92E^{06346}$*/ TM3, Sb* (Bloomington *Drosophila* Stock Center, BL11681), *vasa-GFP knock-in* (from Dr. Tatjana Trcek, Johns Hopkins University, Baltimore, MD).

Spatiotemporally controlled experiments: To study the function of Set1 in the *Drosophila* testis, two RNAi lines *UAS-set1 RNAi* and *UAS-mCherry RNAi* were crossed with different drivers *nos-Gal4/Cyo, bam-Gal4/TM6B*, and *tj-Gal4/Cyo* at 18℃. Newly eclosed progenies were transferred to new vials at 25℃ and aged for 0,1, 3, 5, or 7 days before dissection.

For Set1 function study in the germline stem cells and gonialblast cells, two RNAi lines *UAS-set1 RNAi* and *UAS-mCherry RNAi* were crossed with *nos-Gal4ΔVP16/Cyo; bam-*

bioRxiv preprint doi: https://doi.org/10.1101/2024.02.14.580277; this version posted February 14, 2024. The copyright holder for this preprint (which was not certified by peer review) is the author/funder, who has granted bioRxiv a license to display the preprint in perpetuity. It is made available under aCC-BY 4.0 International license.

Gal80/MKRS at 25℃. Newly eclosed progeny were transferred to new vials and maintained at 25˚for 7 days before dissection.

To determine the role of Set1 in the drosophila early germline at adulthood, two RNAi lines *vasa-GFP knock-in/CyO; UAS-set1 RNAi* and *vasa-GFP knock-in/CyO; UAS-mCherry RNAi* were crossed with *nos-Gal4/Cyo; tub-Gal80^{ts}* at 18℃. Newly eclosed progenies were transferred to new vials at 29℃ and aged for 0, 7, 14, 21, or 28 days before dissection.

To study if expression of *EGFP- set1 cDNA* in the early germline is sufficient to rescue *nos>set1 RNAi* germ cell phenotypes, flies with the following genotypes: *nos-Gal4/ UASp-FRT-EGFP-set1 (WT rescue)-PolyA-FRT; UAS-set1 RNAi/+, nos-Gal4/ UASp-FRT-EGFP-set1 (Mutant rescue)-PolyA-FRT; UAS-set1 RNAi/+, nos-Gal4/ UASp-FRT-EGFP-set1 (WT)-PolyA-FRT; UAS-set1 RNAi/+, nos-Gal4/ UAS- GFP.nls; UAS-set1 RNAi/+* were grown at 25℃ and dissected at 1 and 5 days post eclosion.

To identify if *set1* genetically interacts with *mad* and *stat92E*, the alleles *stat92E^{06346}*, and *mad^{12}* were used. Flies with the following genotypes: *nos-Gal4/+; UAS set1 RNAi/ stat92E^{06346}*, *UAS set1 RNAi/stat92E^{06346}*, *nos-Gal4/mad^{12}; UAS-set1 RNAi/+*, *nos-Gal4/mad^{12}*, and *nos-Gal4/+; UAS set1 RNAi/+* were grown at 25℃ and dissected at 5 days post eclosion.

Generation of transgenic fly lines: For the lines *UASp-FRT-EGFP-set1 (WT rescue)-PolyA-FRT*, *UASp-FRT-EGFP-set1 (Mutant rescue)-PolyA-FRT*, the RNAi recognition sequence was altered. Since the RNAi recognition sequence is in the coding region of the Set1 gene: AAGGTGCAGAGTATAAGAGTA, the third base for each codon of this sequence was changed to make a silent mutation that would allow for the protein to be translated properly but for the RNAi to not recognize the mRNA. For the line *UASp-FRT-EGFP-set1 (Mutant rescue)-PolyA-*

bioRxiv preprint doi: https://doi.org/10.1101/2024.02.14.580277; this version posted February 14, 2024. The copyright holder for this preprint (which was not certified by peer review) is the author/funder, who has granted bioRxiv a license to display the preprint in perpetuity. It is made available under aCC-BY 4.0 International license.

FRT, a mutation in the SET domain at G4713A to make the E1613K amino acid replacement was made.

Immunofluorescence: Testes were dissected in Schneider's insect media and then fixed in 4% formaldehyde in 1X PBST (1X PBS with 0.1% Trition X-100) for 8 min at room temperature (RT). The testes were rinsed three times and washed three times for 5 min each time using 1X PBST at RT. Testes were incubated with primary antibodies in 1X PBST + 3% BSA at 4℃ for at least one night. Samples were then rinsed three times and washed three times for 5 min each time in 1X PBST and then incubated in 1:1,000 dilution of Alexa-Flour conjugated secondary antibody in 5% NGS + PBST for 2 hours at RT or rotating for a minimum of 24 hours at 4℃. Samples were rinsed three times and washed three times, 5 minutes each in 1X PBST and then mounted for microscopy in vectashield antifade mounting medium (Cat#H-1400, Vector laboratories) with or without DAPI. Samples were imaged using a Leica SP8 or Stellaris 5 confocal microscope with a 63x oil immersion objective. Images were analyzed using imageJ software. Primary antibodies used are: Vasa (rabbit, 1:5,000, from R. Lehmann), Fas III (mouse, 1:50, DSHB, 7G10), Armadillo (mouse,1:50, DSHB, N2 7A1), Alpha -spec (mouse, 1:50, DHSB, 3A9), TJ (guinea pig, 1:1000, from M. Van Doren), H3K4me3 (rabbit, 1:400, cell signaling, 9751S), GFP (chicken, 1:1000, Abcam ab13970), Stat92E (rabbit, 1:200, gift from Denise Montell, University of Santa Barbara, CA, USA), and pMad (rabbit, 1:100, Abcam, ab52903).

RNA-seq and data analysis: Flies with the following genotypes: *nos-Gal4; UAS-mCherry RNAi* or *nos-Gal4; UAS-set1 RNAi* were collected as newly eclosed males and aged for 0, 1, 3,

bioRxiv preprint doi: https://doi.org/10.1101/2024.02.14.580277; this version posted February 14, 2024. The copyright holder for this preprint (which was not certified by peer review) is the author/funder, who has granted bioRxiv a license to display the preprint in perpetuity. It is made available under aCC-BY 4.0 International license.

and 5D at 25℃ after shift from 18℃. Approximately 15 pairs of testes for each genotype were dissected in schneiders media + 10% FBS as one replicate. Three replicates were generated for each time point and genotype. The testes were then desheathed in 500 μL of lysis buffer (tryspin LE + 2 mg/ml collagenase). The samples were incubated in lysis buffer for 10 min in a 37℃ water bath with gentle vortex mixing every 2 min. The samples were then filtered through a 40 μm tissue culture filter followed by a 10 min centrifugation at 1,200 rpm. The cells were washed with 200 ul of PBS and pelleted again for 5 min at 1,200 rpm. Total RNA was purified following the manufacturers instruction of the Quick-RNA Microprep Kit (R1050, Zymo Research corporation). The libraries were generated using the reagents provided in NEBNext Ultra II Directional RNA library Prep Kit for Illumina (E7760S, New England Biolabs Inc) and NEB Next Poly(A) mRNA Magnetic Isolation Module (E7490, New England Biolabs inc). The illumina compatible libraries were sequenced with Illumina Novaseq6000 sequencer at the National Institutes of Health sequencing facility.

The sequencing reads were examined using the FastQC quality software (Galaxy Version 0.73+galaxy0) (Andrews). Reads that passed the quality filter were then mapped to the *Drosophila* genome (D. melanogaster Aug 2014 (BDGP Release 6 + ISO11 MT/dm6)) using Bowtie 2 version 2.5.1 (Langmead et al., 2012). For gene mapping, the gene model (Drosophila_melanogaster.BDGP6.87.gtf) was utilized, and we followed an RNA-seq data analysis tutorial using Galaxy, as detailed in the work (Batut et al., 2018; Bérénice Batut; Hiltemann et al., 2023). Aligned reads were summarized to genes using featureCounts (Liao et al., 2014) with gene features obtained from NCBI RefSeq dm6 assembly. Raw counts were normalized to TPM counts. Principal component analysis (PCA) was performed on 16,610 genes with detectable expression in at least one sample, based on which sample distance matrix was

bioRxiv preprint doi: https://doi.org/10.1101/2024.02.14.580277; this version posted February 14, 2024. The copyright holder for this preprint (which was not certified by peer review) is the author/funder, who has granted bioRxiv a license to display the preprint in perpetuity. It is made available under aCC-BY 4.0 International license.

computed. To assess differential gene expression between Set1 knockdown and controls, DESeq2 (Love et al., 2014) was applied on samples from each day, using the Benjamini and Hochberg method for multiple testing correction with default DESeq2 parameters. A threshold of $\log_2$ fold change of 1.3 and adjusted $P < 0.05$ was applied to ascertain differentially expressed genes, for which EnrichR (Chen et al., 2013) was applied to assess enrichment of molecular pathways in each set. All statistical analysis was conducted using R software version 4.2.0.

Phenotype Quantification: All phenotypic quantification was done in Fiji (ImageJ). GSC number was determined by counting every cell that was either Vasa positive or Tj negative and directly next to the hub. Early cyst cell number was quantified by counting every Tj positive cell in a whole testis z stack. Hub area quantification was determined by drawing a line around the z slice with the largest hub size and using the area measurement in Fiji.

Statistics and reproducibility: Data was subjected to the Shapiro-Wilk test to determine whether the data was normally distributed or skewed. For normally distributed data, an unpaired two sample t test was used to compare two individual datasets to each other. For skewed data, the Wilcoxon signed rank test was used to compare two individual datasets to each other. Data are presented with error bars representing the mean ± SE (standard error). Significant differences based on these statistical analysis were noted by asterisks (*$P < 0.05$, **$P < 10^{-2}$, ***$P < 10^{-3}$, ****$P < 10^{-4}$).

bioRxiv preprint doi: https://doi.org/10.1101/2024.02.14.580277; this version posted February 14, 2024. The copyright holder for this preprint (which was not certified by peer review) is the author/funder, who has granted bioRxiv a license to display the preprint in perpetuity. It is made available under aCC-BY 4.0 International license.

Supplemental figures and figure legends:

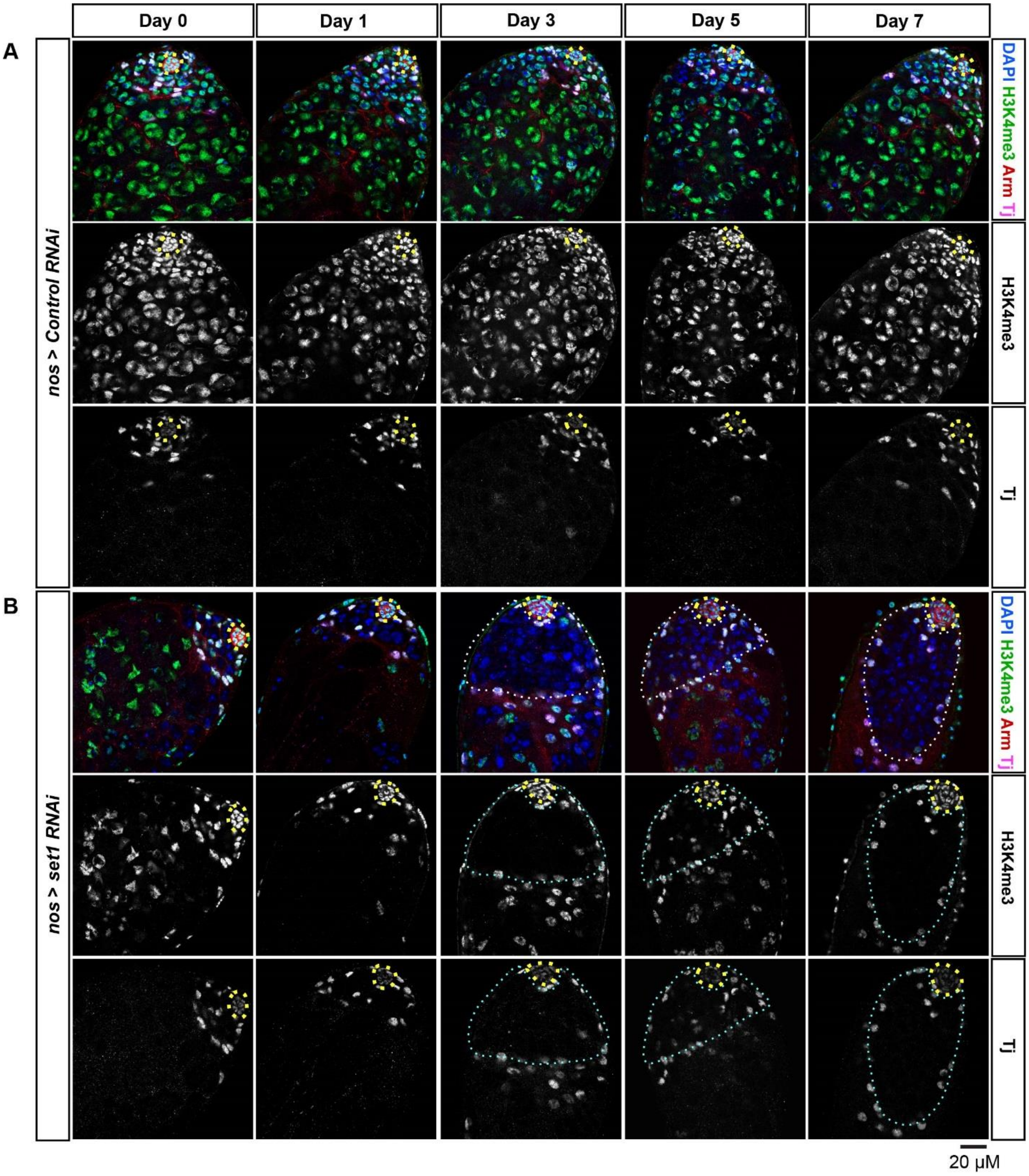

Figure S1: H3K4me3 in the *Drosophila* testis is dependent on Set1. Representative images of *nos>Control RNAi* testes (**A**) and *nos>Set1 RNAi* testes (**B**) at day 0, 1, 3, 5 and 7 post eclosion immunostained with H3K4me3 (green), Arm (red) for the hub region (yellow dotted outline),

bioRxiv preprint doi: https://doi.org/10.1101/2024.02.14.580277; this version posted February 14, 2024. The copyright holder for this preprint (which was not certified by peer review) is the author/funder, who has granted bioRxiv a license to display the preprint in perpetuity. It is made available under aCC-BY 4.0 International license.

and Tj (magenta) for the CySC lineage cells, DAPI (blue). Cyan dotted outlines: overpopulated early-stage germ cells. Scale: 20μm.

bioRxiv preprint doi: https://doi.org/10.1101/2024.02.14.580277; this version posted February 14, 2024. The copyright holder for this preprint (which was not certified by peer review) is the author/funder, who has granted bioRxiv a license to display the preprint in perpetuity. It is made available under aCC-BY 4.0 International license.

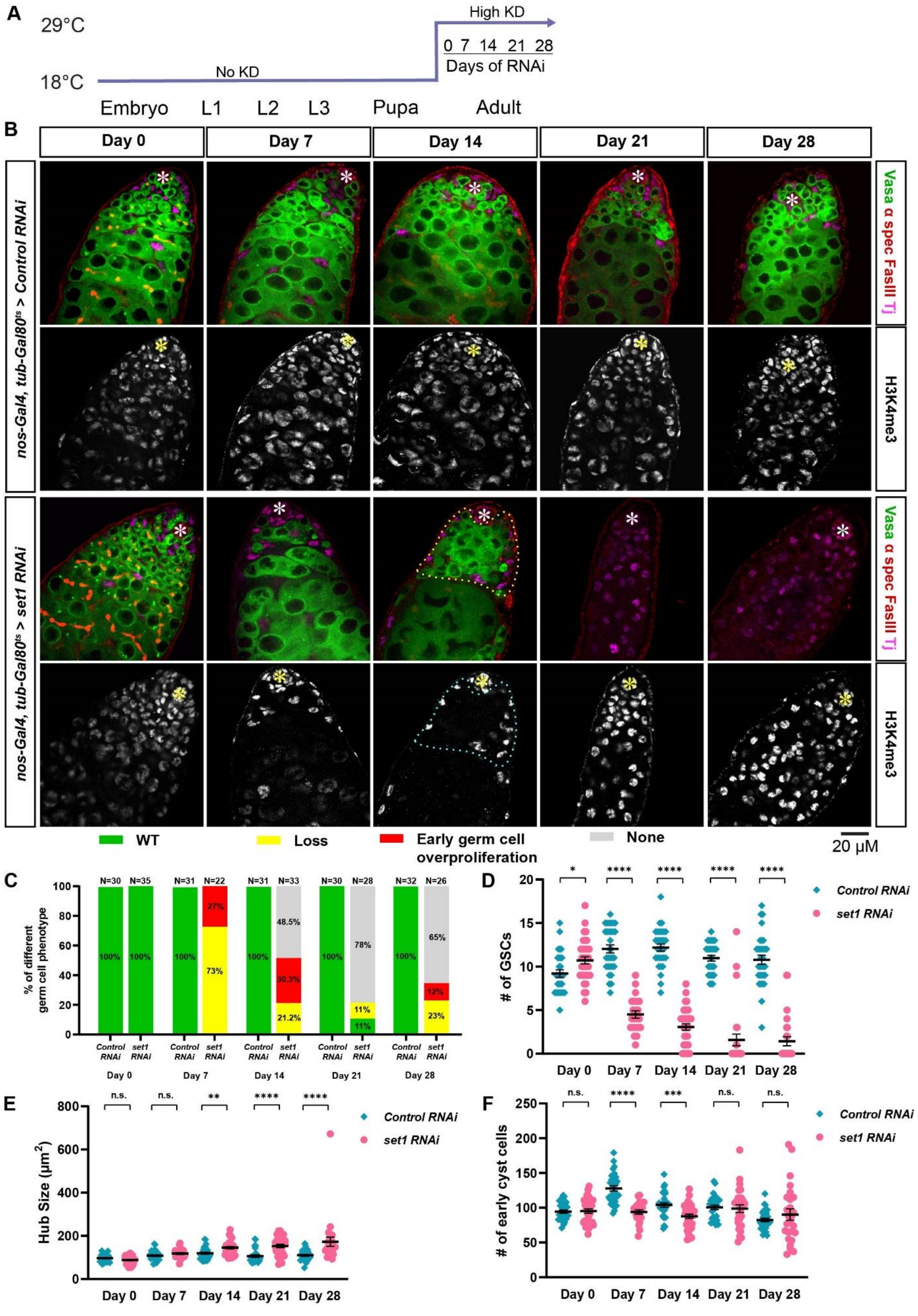

bioRxiv preprint doi: https://doi.org/10.1101/2024.02.14.580277; this version posted February 14, 2024. The copyright holder for this preprint (which was not certified by peer review) is the author/funder, who has granted bioRxiv a license to display the preprint in perpetuity. It is made available under aCC-BY 4.0 International license.

Figure S2: Knockdown of *set1* exclusively in the adult *Drosophila* testis leads to germ cell loss and germline differentiation defects. (**A**) *tub-Gal80^{ts}, nos>Control RNAi* and *tub-Gal80^{ts}, nos>set1 RNAi* flies are grown at the permissive temperature (18℃) until eclosion, and shifted to the restrictive temperature (29˚C) for 0, 7, 14, 21, and 28 days, respectively. (**B**) Representative images of *tub-Gal80^{ts}, nos>Control RNAi* and *tub-Gal80^{ts}, nos>set1 RNAi* testes at day 0, 7, 14, 21 and 28 post eclosion, *vasa-GFP* from a knock-in strain label the germline, immunostained with H3K4me3 (grey), Fas III (red) for the hub region, and Tj (magenta) for the CySC lineage cells. Asterisk: hub. White dotted outlines: overpopulated early-stage germ cells. (**C-D**) Quantification of the percentage of testes with the germline phenotypes (**C**) and GSC number (**D**) in *tub-Gal80^{ts}, nos>set1 RNAi* and *tub-Gal80^{ts}, nos>Control RNAi* testes at 0, 7, 14, 21 and 28 days post eclosion, respectively**:** *tub-Gal80^{ts}, nos>Control RNAi* testes (Day 0: N=30; Day 7: N=31; Day 14: N=31; Day 21: N=30; Day 28: N=32) and *tub-Gal80^{ts}, nos>set1 RNAi* testes (Day 0: N=35; Day 7: N=22; Day 14: N=33; Day 21: N=28; Day 28: N=26). Refer to Table S1. (**E**) Quantification of hub region size for *tub-Gal80^{ts}, nos>Control RNAi* and *tub-Gal80^{ts}, nos>set1 RNAi* testes. Refer to Table S2. (**F**) Quantification of early cyst cell number for *tub-Gal80^{ts}, nos>Control RNAi* and *tub-Gal80^{ts}, nos>set1 RNAi* testes. Refer to Table S3. Individual data points and mean values are shown. Error bars represent SEM. ****$P< 10^{-4}$, ***$P< 10^{-3}$, **$P< 10^{-2}$, *$P< 0.05$, n.s.: not significant; unpaired t test to compare two individual datasets with each other.

bioRxiv preprint doi: https://doi.org/10.1101/2024.02.14.580277; this version posted February 14, 2024. The copyright holder for this preprint (which was not certified by peer review) is the author/funder, who has granted bioRxiv a license to display the preprint in perpetuity. It is made available under aCC-BY 4.0 International license.

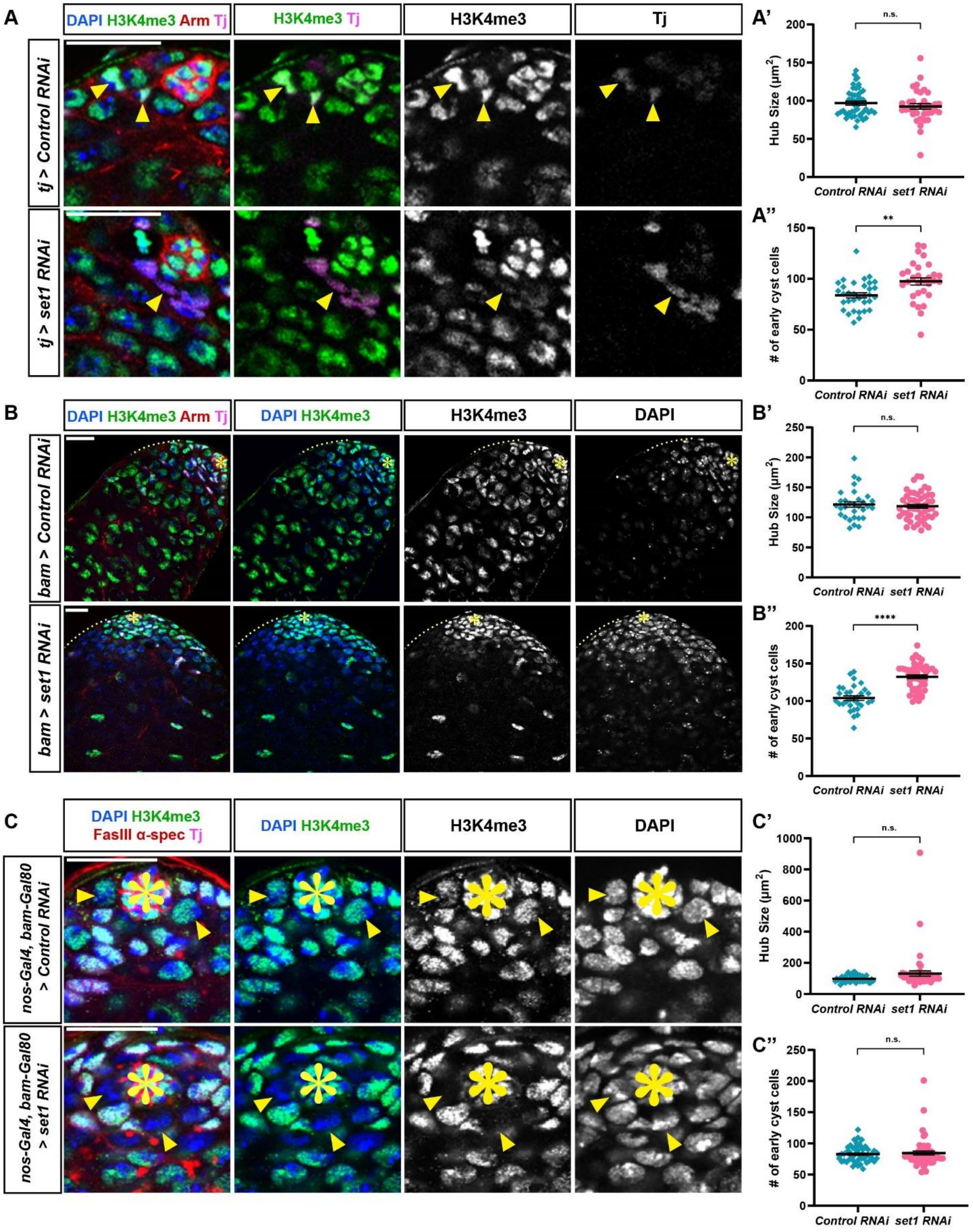

bioRxiv preprint doi: https://doi.org/10.1101/2024.02.14.580277; this version posted February 14, 2024. The copyright holder for this preprint (which was not certified by peer review) is the author/funder, who has granted bioRxiv a license to display the preprint in perpetuity. It is made available under aCC-BY 4.0 International license.

Figure S3: H3K4me3 signals are reduced in the somatic gonadal cells, late spermatogonial cells, and GSCs in the *Drosophila* testis by knockdown of *set1* using cell type- and stage-specific drivers. (**A**) Representative images of *tj>Control RNAi* and *tj>set1 RNAi* testes at day 7 post eclosion, immunostained with H3K4me3 (green), Arm (red) for the hub region, and Tj (magenta) for the CySC lineage cells. (**A'**) Quantification of the hub region size for *tj>Control RNAi* testes (N= 30) and *tj>set1 RNAi* testes (N= 30). Refer to Table S2. (**A''**) Quantification of cyst cell number for *tj>Control RNAi* and *tj>set1 RNAi* testes. Individual data points and mean values are shown. Refer to Table S3. (**B**) Representative images of *bam>Control RNAi* and *bam>set1 RNAi* testes at day 7 post eclosion, immunostained with H3K4me3 (green), Arm (red) for the hub region, and Tj (magenta) for the CySC lineage cells. (**B'**) Quantification of the hub size for *bam>Control RNAi* testes (N= 31) and *bam>set1 RNAi* testes (N= 50). Refer to Table S2. (**B''**) Quantification of cyst cell number for *bam>Control RNAi* and *bam>set1 RNAi* testes. Refer to Table S3. (**C**) Representative images of *nos-Gal4ΔVP16, bam-Gal80 >Control RNAi* and *nos-Gal4ΔVP16, bam-Gal80 >set1 RNAi* testes at day 7 post eclosion, immunostained with H3K4me3 (green), Arm (red) for the hub region, and Tj (magenta) for the CySC lineage cells. (**C'**) Quantification of hub region size for *nos-Gal4ΔVP16, bam-Gal80 >Control RNAi* testes (N= 52) and *nos-Gal4ΔVP16, bam-Gal80 >set1 RNAi* testes (N= 52). Refer to Table S2. (**C''**) Quantification of cyst cell number for *nos-Gal4ΔVP16, bam-Gal80>Control RNAi* and *nos-Gal4ΔVP16, bam-Gal80>set1 RNAi* testes. Refer to Table S3. (**A'-A", B'-B", C'-C"**) Individual data points and mean values are shown. Error bars represent SEM. ****$P< 10^{-4}$, **$P< 10^{-2}$, n.s.: not significant, unpaired t test to compare two individual datasets to each other. Asterisk in (**B** and **C**): Hub region. Yellow arrow heads: cyst cells in (**A**); GSCs in (**C**). Yellow dotted line: 4-16 spermatogonial cell region in (**B**). Scale: 20 μm.

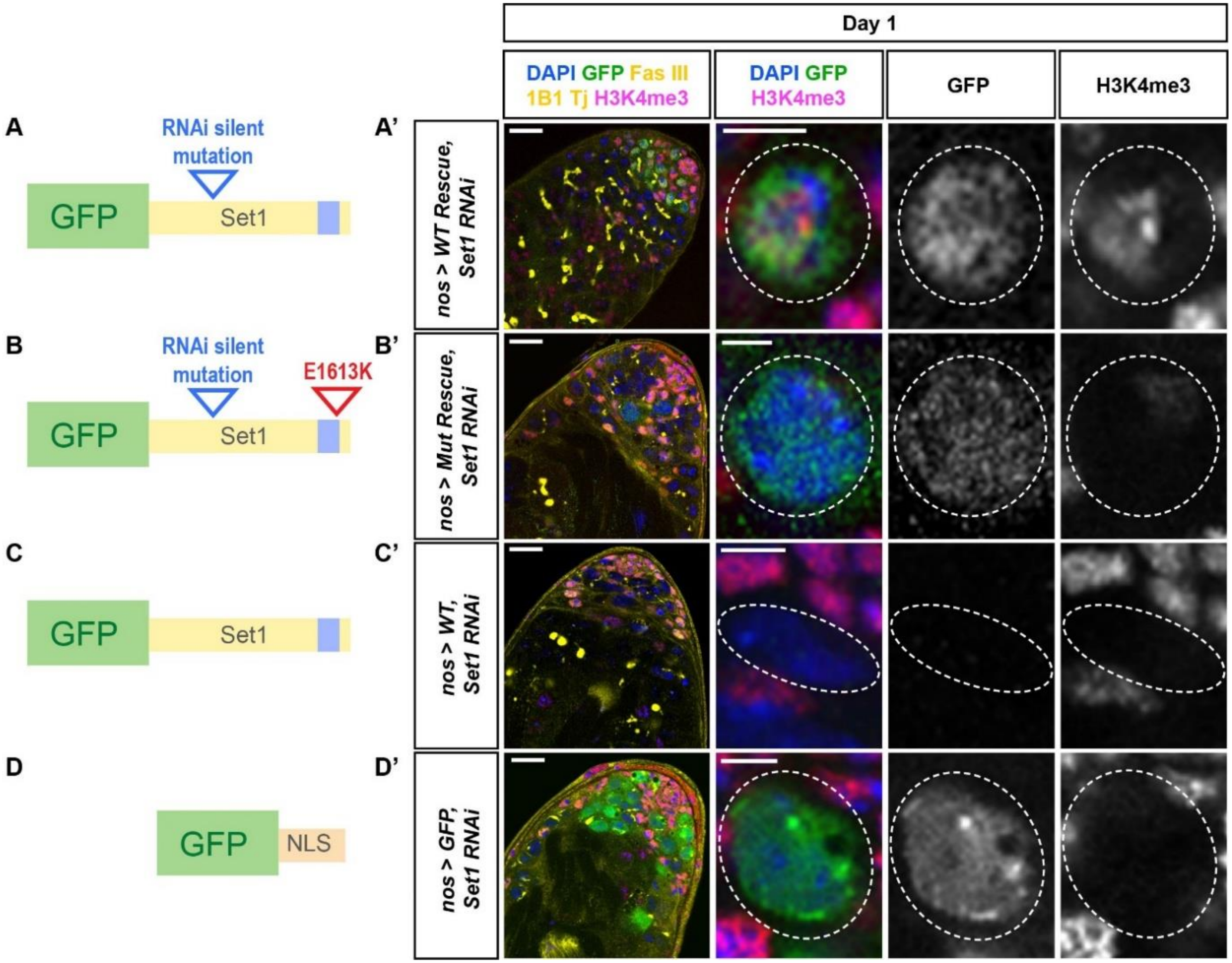
Day 1
DAPI GFP Fas III 1B1 Tj H3K4me3
DAPI GFP H3K4me3
GFP
H3K4me3
A
RNAi silent mutation
GFP
Set1
A'
nos > WT Rescue, Set1 RNAi
B
RNAi silent mutation
E1613K
GFP
Set1
B'
nos > Mut Rescue, Set1 RNAi
C
GFP
Set1
C'
nos > WT, Set1 RNAi
D
GFP
NLS
D'
nos > GFP, Set1 RNAi

bioRxiv preprint doi: https://doi.org/10.1101/2024.02.14.580277; this version posted February 14, 2024. The copyright holder for this preprint (which was not certified by peer review) is the author/funder, who has granted bioRxiv a license to display the preprint in perpetuity. It is made available under aCC-BY 4.0 International license.

Figure S4: The methyltransferase activity of Set1 is required for its function in the male germline. (**A**-**D**) Cartoon depictions: the *set1* cDNA transgene with the RNAi recognition sequences mutated, named WT rescue (**A**); the *set1* cDNA transgene with the RNAi recognition sequences mutated and an E→K amino acid change in the SET domain, named Mut rescue (**B**); the *set1* cDNA transgene without the RNAi recognition sequences mutated, named WT (**C**); the *GFP* cDNA with the *nuclear localization* sequence, named GFP (**D**). Representative images of *nos-Gal4>WT Rescue, set1 RNAi* testis (**A'**), *nos-Gal4>Mut Rescue, set1 RNAi* testis (**B'**), *nos-Gal4>WT, set1 RNAi* testis (**C'**), *nos-Gal4>GFP, set1 RNAi* testis (**D'**), all testes are from males 1 day post eclosion, immunostained with H3K4me3 (magenta), GFP (green), Fas III (yellow) for the hub region, and Tj (yellow) for the CySC lineage cells. White dotted outline: germ cell expressing the corresponding transgenes. Scale: 20 μm for the testis image; 5 μm for individual germ cell images.

bioRxiv preprint doi: https://doi.org/10.1101/2024.02.14.580277; this version posted February 14, 2024. The copyright holder for this preprint (which was not certified by peer review) is the author/funder, who has granted bioRxiv a license to display the preprint in perpetuity. It is made available under aCC-BY 4.0 International license.

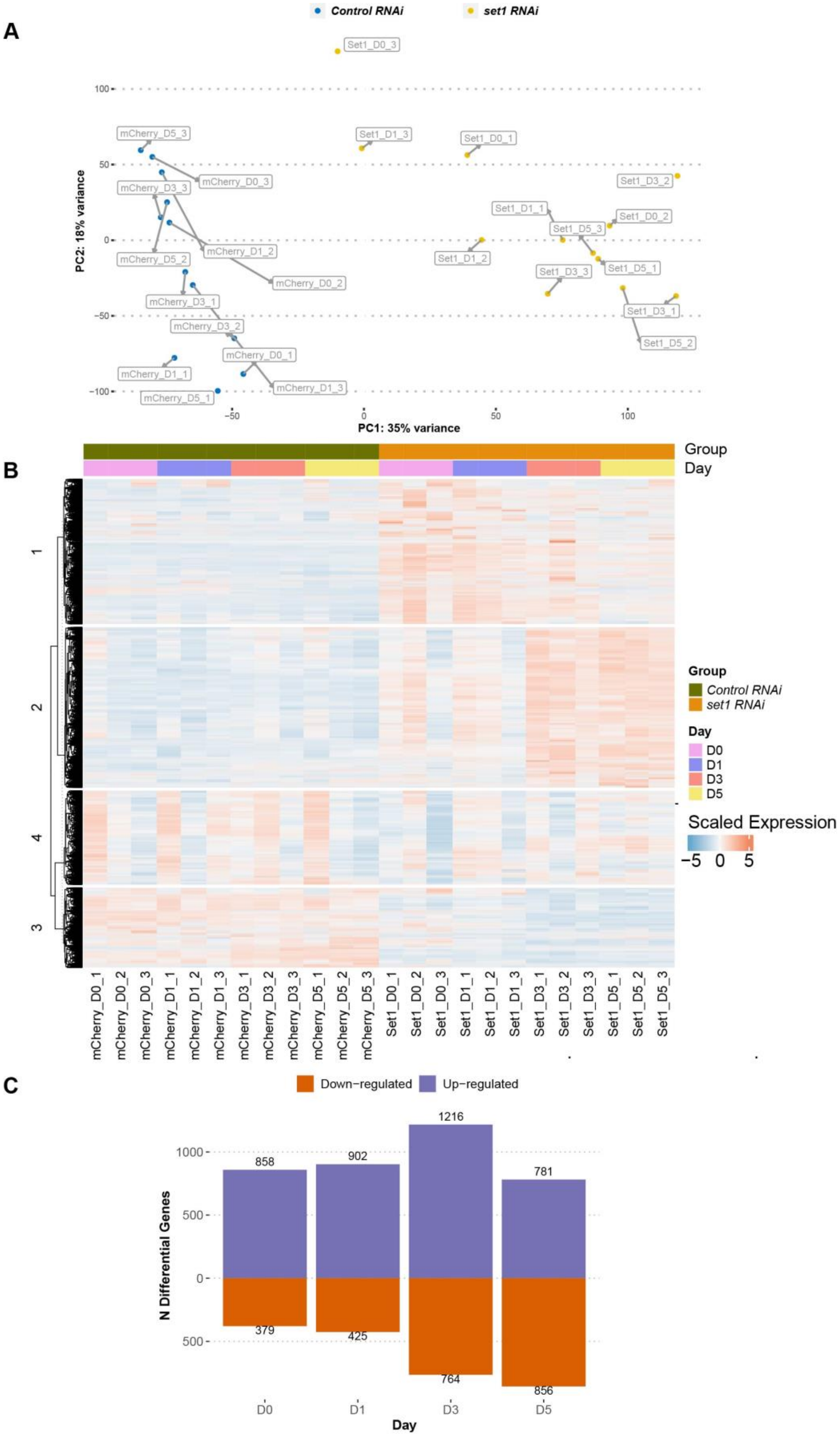

bioRxiv preprint doi: https://doi.org/10.1101/2024.02.14.580277; this version posted February 14, 2024. The copyright holder for this preprint (which was not certified by peer review) is the author/funder, who has granted bioRxiv a license to display the preprint in perpetuity. It is made available under aCC-BY 4.0 International license.

D

Gene	Log2 FC	Adjusted P-value
JAK-STAT signaling pathway		
upd1	-0.85	2.82E-04
upd2	1.26	0.06
hop	0.52	0.03
dome	0.16	0.49
BMP signaling pathway		
gbb	0.70	4.45E-03
mad	0.03	0.89
med	0.80	6.14E-05
put	1.26	4.86E-09
sax	-0.29	0.20
tkv	0.62	4.89E-03

bioRxiv preprint doi: https://doi.org/10.1101/2024.02.14.580277; this version posted February 14, 2024. The copyright holder for this preprint (which was not certified by peer review) is the author/funder, who has granted bioRxiv a license to display the preprint in perpetuity. It is made available under aCC-BY 4.0 International license.

Figure S5: Knockdown of *set1* in the early germline results in global gene expression changes. (**A**) Principal component analysis plot shows multidimensional distribution of all *nos>set1 RNAi* (*set1* KD, 12 samples) and *nos>Control RNAi* (*Ctrl* KD, 12 samples) data sets: Three biological replicates for both *set1* KD and *Ctrl* KD at day 0, 1, 3, 5 post eclosion, respectively. There are two main clusters: a *set1* KD cluster and a *Ctrl* KD cluster. Within the *set1* KD cluster, Day 0 and Day 1 timepoint samples cluster while Day 3 and Day 5 timepoint samples cluster. (**B**) Heatmap shows differentially expressed genes (DEGs) between *set1* KD and *Ctrl* KD testes at day 0, 1, 3, 5 post eclosion, respectively. All genes are separated into four different groups. Group 1 contains the genes that are most expressed in *set1* KD testes at Day 0 and 1. Group 2 consists of the genes that are most expressed in *set1* KD testes at Day 3 and 5. Group 3 is comprised of genes more highly expressed in *Ctrl* KD testes and Group 4 is composed of the genes that did not fit any particular expression pattern with regards to genotype or timepoint. (**C**) Bar plot shows the number of DEGs with ≥1.3-fold ($\log_2$ scale) and $P< 0.05$ in *set1* KD testes compared to *Ctrl* KD testes at day 0, 1, 3, 5 post eclosion, respectively. (**D**) A table shows the detailed expression changes of several core components of the JAK-STAT and BMP signaling pathways at Day 3. Genes that are significantly upregulated are highlighted in purple and genes that were significantly downregulated are highlighted in orange.

bioRxiv preprint doi: https://doi.org/10.1101/2024.02.14.580277; this version posted February 14, 2024. The copyright holder for this preprint (which was not certified by peer review) is the author/funder, who has granted bioRxiv a license to display the preprint in perpetuity. It is made available under aCC-BY 4.0 International license.

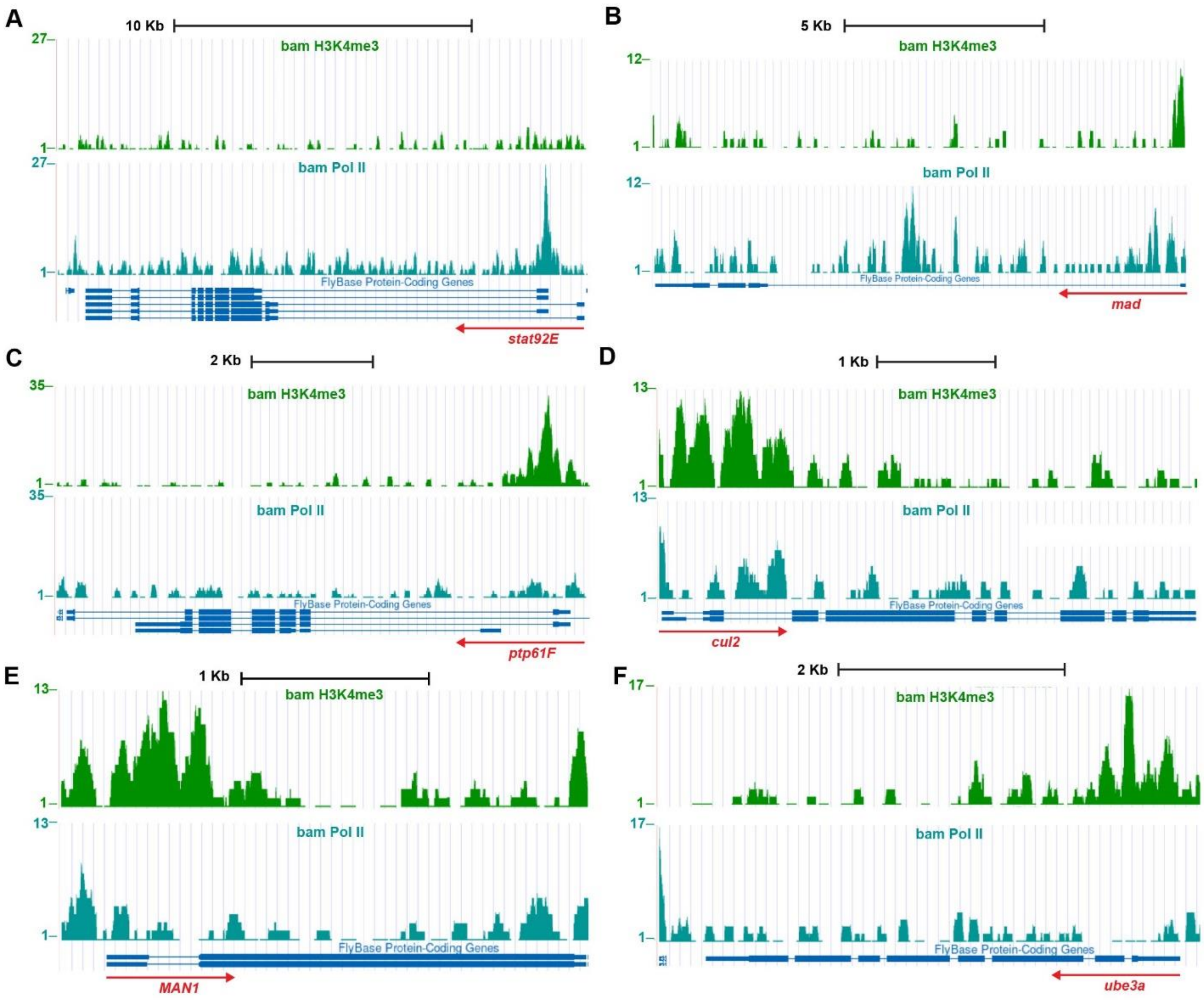

G

Gene	Log2 FC	Adjusted P-value
Negative regulators of JAK-STAT signaling pathway		
ptp61F	-0.14	0.51
Negative regulators of BMP signaling pathway		
cul2	-0.15	0.43
MAN1	-0.35	0.08
ube3a	-0.04	0.86

bioRxiv preprint doi: https://doi.org/10.1101/2024.02.14.580277; this version posted February 14, 2024. The copyright holder for this preprint (which was not certified by peer review) is the author/funder, who has granted bioRxiv a license to display the preprint in perpetuity. It is made available under aCC-BY 4.0 International license.

Figure S6: UCSC genome browser screenshots show H3K4me3 and RNA Pol II enrichment at individual gene regions. (**A**-**F**) All data are based on ChIP-seq using progenitor germ cell-enriched *bam* mutant testes (Gan et al., 2010), H3K4me3 (green) and RNA polymerase II (blue). (**A**) At the *stat92E* gene locus, H3K4me3 is not distinctly enriched at the promoter region even though Pol II profiles indicates active transcription. (**B**) At the *mad* gene locus, H3K4me3 is enriched at the promoter region and Pol II profiles indicates active transcription. (**C**) At the *ptp61F* gene locus, H3K4me3 is very distinctly enriched at the promoter region, even though Pol II profiles indicates inactive transcription. (**D**) At the *cul-2* gene locus, H3K4me3 is enriched at the promoter region and Pol II profiles indicates active transcription. (**E**) At the *MAN1* gene locus, H3K4me3 is enriched at the promoter region and Pol II profiles indicates active transcription. (**F**) At the *ube3a* gene locus, H3K4me3 is very distinctly enriched at the promoter region, even though Pol II profiles indicates inactive transcription. (**G**) A table shows the detailed expression changes of several inhibitors of the JAK-STAT and BMP signaling pathways at Day 3.

bioRxiv preprint doi: https://doi.org/10.1101/2024.02.14.580277; this version posted February 14, 2024. The copyright holder for this preprint (which was not certified by peer review) is the author/funder, who has granted bioRxiv a license to display the preprint in perpetuity. It is made available under aCC-BY 4.0 International license.

Supplemental Tables

Table S1: Quantification of Germline Stem Cell number in RNAi knockdown testes.

nanos driven Control RNAi Data:

Testis #	Day 0	Day 1	Day 3	Day 5	Day 7
1	11	7	12	11	9
2	12	11	14	8	12
3	11	13	9	14	13
4	15	12	11	8	8
5	18	9	10	13	12
6	10	12	14	11	7
7	11	11	11	15	13
8	14	11	12	11	9
9	15	8	14	9	13
10	14	13	12	13	11
11	14	13	13	12	11
12	12	12	10	10	10
13	14	13	15	9	11
14	14	10	13	14	11
15	10	7	14	12	14
16	10	13	11	18	7
17	12	10	7	12	12
18	13	15	15	15	12

bioRxiv preprint doi: https://doi.org/10.1101/2024.02.14.580277; this version posted February 14, 2024. The copyright holder for this preprint (which was not certified by peer review) is the author/funder, who has granted bioRxiv a license to display the preprint in perpetuity. It is made available under aCC-BY 4.0 International license.

19	15	12	12	17	14
20	13	11	13	15	12
21	12	13	12	12	9
22	15	11	15	13	10
23	11	12	14	9	11
24	11	15	12	11	11
25	13	11	11	13	8
26	13	12	13	11	13
27	11	9	9	10	12
28	14	12	15	8	9
29	13	11	10	8	11
30	15	8	9	9	
31	10	11		10	
32	10	13			
33	10	11			
34	14	10			
35	14	10			
36		9			
37		9			
38		9			
39		16			
40		9			

bioRxiv preprint doi: https://doi.org/10.1101/2024.02.14.580277; this version posted February 14, 2024. The copyright holder for this preprint (which was not certified by peer review) is the author/funder, who has granted bioRxiv a license to display the preprint in perpetuity. It is made available under aCC-BY 4.0 International license.

nanos driven Set1 RNAi Data:

Testis #	Day 0	Day 1	Day 3	Day 5	Day 7
1	4	7	7	19	6
2	6	2	7	9	10
3	6	5	4	9	9
4	6	12	12	7	10
5	10	9	17	10	7
6	4	12	10	8	6
7	11	7	10	6	6
8	9	6	9	5	16
9	12	9	8	8	7
10	10	8	7	5	9
11	8	7	17	9	4
12	3	18	14	5	7
13	14	4	11	5	8
14	9	9	9	15	14
15	5	10	10	7	10
16	6	5	10	16	8
17	10	9	8	16	10
18	10	2	9	9	11
19	4	11	15	7	9
20	11	7	10	9	9

bioRxiv preprint doi: https://doi.org/10.1101/2024.02.14.580277; this version posted February 14, 2024. The copyright holder for this preprint (which was not certified by peer review) is the author/funder, who has granted bioRxiv a license to display the preprint in perpetuity. It is made available under aCC-BY 4.0 International license.

21	7	7	14	11	14
22	5	7	4	11	16
23	11	9	8	13	12
24	7	6	8	8	10
25	8	5	12	9	5
26	11	7	11	9	23
27	13	12	9	9	6
28	10	5	10	10	5
29	12	14	12	14	7
30	6	8	7	13	12
31	8	8	7	8	6
32	10	10	7	8	6
33	8	5	3	12	8
34	9	6		16	5
35	12			9	5
36	6			6	6
37	6			15	7
38	2			12	13
39	7			16	6
40	4			8	
41	3				
42	6				

bioRxiv preprint doi: https://doi.org/10.1101/2024.02.14.580277; this version posted February 14, 2024. The copyright holder for this preprint (which was not certified by peer review) is the author/funder, who has granted bioRxiv a license to display the preprint in perpetuity. It is made available under aCC-BY 4.0 International license.

43	6				
44	5				
45	3				
46	3				
47	5				
48	7				
49	5				
50	2				
51	3				

nos-gal4, tub-gal80^{ts} driven Control RNAi Data:

**Testis #**	**Day 0**	**Day 7**	**Day 14**	**Day 21**	**Day 28**
1	7	11	9	10	9
2	7	13	12	10	12
3	9	14	14	12	10
4	9	15	13	9	12
5	12	11	12	13	10
6	7	11	14	12	11
7	10	12	18	13	13
8	12	10	13	12	8
9	8	14	12	14	12
10	7	15	11	13	16

bioRxiv preprint doi: https://doi.org/10.1101/2024.02.14.580277; this version posted February 14, 2024. The copyright holder for this preprint (which was not certified by peer review) is the author/funder, who has granted bioRxiv a license to display the preprint in perpetuity. It is made available under aCC-BY 4.0 International license.

11	8	11	13	8	9
12	7	16	14	12	8
13	12	12	11	9	9
14	7	15	7	9	3
15	7	10	11	12	11
16	8	10	10	10	12
17	11	14	11	12	6
18	8	14	10	10	13
19	11	14	11	10	13
20	15	8	15	12	12
21	9	9	14	12	17
22	9	11	13	13	12
23	11	8	8	13	10
24	11	15	14	9	8
25	5	10	11	11	8
26	10	15	13	8	9
27	14	15	14	9	10
28	9	9	15	13	12
29	7	7	10	11	16
30	9	14	12	8	15
31		10	13		10
32					9

bioRxiv preprint doi: https://doi.org/10.1101/2024.02.14.580277; this version posted February 14, 2024. The copyright holder for this preprint (which was not certified by peer review) is the author/funder, who has granted bioRxiv a license to display the preprint in perpetuity. It is made available under aCC-BY 4.0 International license.

nos-gal4, tub-gal80^{ts} driven Set1 RNAi Data:

Testis #	Day 0	Day 7	Day 14	Day 21	Day 28
1	12	5	4	0	0
2	6	6	0	0	2
3	12	6	1	0	0
4	11	2	7	0	3
5	10	3	1	0	0
6	11	7	3	0	3
7	17	8	0	0	0
8	11	4	4	3	0
9	7	9	4	0	0
10	9	3	3	0	5
11	9	4	2	0	0
12	12	3	6	0	0
13	12	3	4	3	0
14	10	4	1	3	0
15	13	6	1	0	2
16	9	3	8	0	4
17	10	6	3	10	0
18	14	2	6	14	0
19	8	4	5	0	9
20	13	5	5	0	0

bioRxiv preprint doi: https://doi.org/10.1101/2024.02.14.580277; this version posted February 14, 2024. The copyright holder for this preprint (which was not certified by peer review) is the author/funder, who has granted bioRxiv a license to display the preprint in perpetuity. It is made available under aCC-BY 4.0 International license.

21	10	1	4	1	9
22	9	5	4	1	0
23	12		0	0	0
24	7		5	9	0
25	14		3	0	0
26	9		4	0	0
27	11		2	0	
28	11		0	0	
29	10		5		
30	15		4		
31	8		0		
32	9		0		
33	9		2		
34	13				
35	12				

tj driven RNAi Data:

**Testis #**	***Control RNAi***	***Set1 RNAi***
1	11	10
2	6	9
3	9	9
4	5	7

bioRxiv preprint doi: https://doi.org/10.1101/2024.02.14.580277; this version posted February 14, 2024. The copyright holder for this preprint (which was not certified by peer review) is the author/funder, who has granted bioRxiv a license to display the preprint in perpetuity. It is made available under aCC-BY 4.0 International license.

5	2	8
6	7	9
7	7	10
8	12	8
9	9	6
10	8	3
11	5	13
12	7	9
13	6	6
14	3	12
15	6	10
16	5	8
17	10	12
18	10	7
19	6	10
20	7	9
21	7	13
22	7	10
23	9	10
24	8	10
25	6	9
26	11	11

bioRxiv preprint doi: https://doi.org/10.1101/2024.02.14.580277; this version posted February 14, 2024. The copyright holder for this preprint (which was not certified by peer review) is the author/funder, who has granted bioRxiv a license to display the preprint in perpetuity. It is made available under aCC-BY 4.0 International license.

27	9	11
28	10	12
29	5	11
30	10	12
31	12	7
32	10	10
33	12	12
34	5	7
35	13	9
36	6	11
37	6	
38	5	
39	5	
40	7	
41	6	
42	5	
43	5	
44	12	
45	11	
46	9	

bam driven RNAi Data:

bioRxiv preprint doi: https://doi.org/10.1101/2024.02.14.580277; this version posted February 14, 2024. The copyright holder for this preprint (which was not certified by peer review) is the author/funder, who has granted bioRxiv a license to display the preprint in perpetuity. It is made available under aCC-BY 4.0 International license.

**Testis #**	***Control RNAi***	***Set1 RNAi***
1	9	7
2	10	9
3	14	15
4	8	6
5	10	14
6	7	10
7	8	11
8	9	9
9	8	8
10	6	8
11	14	11
12	12	7
13	8	7
14	8	10
15	9	11
16	15	6
17	9	12
18	9	6
19	13	10
20	8	12
21	7	9

bioRxiv preprint doi: https://doi.org/10.1101/2024.02.14.580277; this version posted February 14, 2024. The copyright holder for this preprint (which was not certified by peer review) is the author/funder, who has granted bioRxiv a license to display the preprint in perpetuity. It is made available under aCC-BY 4.0 International license.

22	7	9
23	12	8
24	10	7
25	12	7
26	15	10
27	12	8
28	4	7
29	5	6
30	10	10
31	9	11
32		14
33		7
34		11
35		8
36		10
37		8
38		11
39		10
40		7
41		9
42		9
43		8

bioRxiv preprint doi: https://doi.org/10.1101/2024.02.14.580277; this version posted February 14, 2024. The copyright holder for this preprint (which was not certified by peer review) is the author/funder, who has granted bioRxiv a license to display the preprint in perpetuity. It is made available under aCC-BY 4.0 International license.

44		11
45		8
46		13
47		10
48		11
49		9
50		8

nos-gal4, bam-gal80 driven RNAi Data:

**Testis #**	***Control RNAi***	***Set1 RNAi***
1	7	6
2	8	7
3	9	7
4	11	6
5	8	7
6	4	7
7	8	8
8	7	6
9	11	6
10	8	7
11	8	6
12	8	4

bioRxiv preprint doi: https://doi.org/10.1101/2024.02.14.580277; this version posted February 14, 2024. The copyright holder for this preprint (which was not certified by peer review) is the author/funder, who has granted bioRxiv a license to display the preprint in perpetuity. It is made available under aCC-BY 4.0 International license.

13	9	6
14	5	6
15	6	2
16	8	5
17	8	4
18	7	6
19	10	8
20	8	6
21	7	5
22	8	3
23	8	9
24	7	4
25	9	5
26	6	7
27	8	6
28	6	6
29	5	8
30	8	7
31	7	6
32	4	6
33	9	6
34	7	6

bioRxiv preprint doi: https://doi.org/10.1101/2024.02.14.580277; this version posted February 14, 2024. The copyright holder for this preprint (which was not certified by peer review) is the author/funder, who has granted bioRxiv a license to display the preprint in perpetuity. It is made available under aCC-BY 4.0 International license.

35	7	11
36	7	5
37	5	3
38	8	2
39	7	2
40	7	4
41	7	6
42	7	1
43	5	4
44	7	3
45	11	3
46	5	3
47	4	3
48	7	3
49	5	4
50	8	3
51	7	6
52	12	6

Mad genetic interaction:

**Testis #**	***mad*^{12}*/ +***	***nos> set1 RNAi***	***mad*^{12}*/ +; nos> Set1 RNAi***
1	7	4	14

bioRxiv preprint doi: https://doi.org/10.1101/2024.02.14.580277; this version posted February 14, 2024. The copyright holder for this preprint (which was not certified by peer review) is the author/funder, who has granted bioRxiv a license to display the preprint in perpetuity. It is made available under aCC-BY 4.0 International license.

2	9	8	6
3	8	7	7
4	9	7	7
5	10	8	8
6	10	6	8
7	11	10	10
8	10	7	7
9	9	13	10
10	9	14	14
11	8	7	8
12	10	5	9
13	9	7	8
14	12	0	6
15	14	4	7
16	11	1	9
17	6	7	9
18	10	9	16
19	9	7	8
20	8	8	13
21	7	10	9
22	9	0	8
23	10	13	5

bioRxiv preprint doi: https://doi.org/10.1101/2024.02.14.580277; this version posted February 14, 2024. The copyright holder for this preprint (which was not certified by peer review) is the author/funder, who has granted bioRxiv a license to display the preprint in perpetuity. It is made available under aCC-BY 4.0 International license.

24	6	0	6
25	8	8	5
26	7	7	9
27	9	8	8
28	9	2	0
29	7	6	9
30	4	6	14
31	10	18	13
32	6	7	8
33	8	4	12
34	9	6	7
35	9	8	7
36	8	8	11
37	11	6	5
38	6	6	11
39	8	10	17
40	8	3	18
41	10	1	0
42	11	3	10
43	8	4	16
44	11	6	
45	4	6	

bioRxiv preprint doi: https://doi.org/10.1101/2024.02.14.580277; this version posted February 14, 2024. The copyright holder for this preprint (which was not certified by peer review) is the author/funder, who has granted bioRxiv a license to display the preprint in perpetuity. It is made available under aCC-BY 4.0 International license.

46	8	7	
47	6	5	
48	6	11	
49	6	0	
50	8	6	
51	5	4	
52	7	12	
53	10	4	
54	4	8	
55	7	6	
56	11	7	
57		9	
58		5	
59		9	
60		4	
61		5	
62		3	
63		7	
64		10	
65		9	
66		1	
67		0	

bioRxiv preprint doi: https://doi.org/10.1101/2024.02.14.580277; this version posted February 14, 2024. The copyright holder for this preprint (which was not certified by peer review) is the author/funder, who has granted bioRxiv a license to display the preprint in perpetuity. It is made available under aCC-BY 4.0 International license.

68		4	
69		0	
70		5	
71		7	
72		2	
73		6	
74		0	
75		5	
76		4	
77		8	
78		9	
79		3	
80		7	
81		6	
82		3	
83		3	
84		3	

Stat92E genetic interaction:

**Testis #**	***stat92E^{06346}/ +***	***nos> set1 RNAi***	***nos> Set1 RNAi; stat92E^{06346}/ +***
1	8	4	8
2	7	8	7

bioRxiv preprint doi: https://doi.org/10.1101/2024.02.14.580277; this version posted February 14, 2024. The copyright holder for this preprint (which was not certified by peer review) is the author/funder, who has granted bioRxiv a license to display the preprint in perpetuity. It is made available under aCC-BY 4.0 International license.

3	13	7	10
4	8	7	7
5	8	8	4
6	5	6	10
7	11	10	10
8	10	7	9
9	10	13	13
10	6	14	10
11	10	7	11
12	9	5	4
13	14	7	4
14	10	0	9
15	9	4	11
16	9	1	11
17	9	7	7
18	8	9	5
19	9	7	6
20	13	8	6
21	10	10	11
22	9	0	7
23	9	13	4
24	10	0	6

bioRxiv preprint doi: https://doi.org/10.1101/2024.02.14.580277; this version posted February 14, 2024. The copyright holder for this preprint (which was not certified by peer review) is the author/funder, who has granted bioRxiv a license to display the preprint in perpetuity. It is made available under aCC-BY 4.0 International license.

25	13	8	9
26	12	7	9
27	6	8	8
28	12	2	13
29	14	6	14
30	11	6	11
31	10	18	0
32	8	7	5
33	9	4	6
34	12	6	10
35	9	8	4
36	11	8	4
37	11	6	10
38	9	6	9
39	11	10	
40	14	3	
41		1	
42		3	
43		4	
44		6	
45		6	
46		7	

bioRxiv preprint doi: https://doi.org/10.1101/2024.02.14.580277; this version posted February 14, 2024. The copyright holder for this preprint (which was not certified by peer review) is the author/funder, who has granted bioRxiv a license to display the preprint in perpetuity. It is made available under aCC-BY 4.0 International license.

47		5	
48		11	
49		0	
50		6	
51		4	
52		12	
53		4	
54		8	
55		6	
56		7	
57		9	
58		5	
59		9	
60		4	
61		5	
62		3	
63		7	
64		10	
65		9	
66		1	
67		0	
68		4	

bioRxiv preprint doi: https://doi.org/10.1101/2024.02.14.580277; this version posted February 14, 2024. The copyright holder for this preprint (which was not certified by peer review) is the author/funder, who has granted bioRxiv a license to display the preprint in perpetuity. It is made available under aCC-BY 4.0 International license.

69		0	
70		5	
71		7	
72		2	
73		6	
74		0	
75		5	
76		4	
77		8	
78		9	
79		3	
80		7	
81		6	
82		3	
83		3	
84		3	

Table S2: Quantification of Hub area in RNAi knockdown testes.

nanos driven Control RNAi Data:

Testis #	Day 0	Day 1	Day 3	Day 5	Day 7
1	115.818	147.691	125.508	141.853	140.686
2	164.736	150.493	148.625	141.269	164.503

bioRxiv preprint doi: https://doi.org/10.1101/2024.02.14.580277; this version posted February 14, 2024. The copyright holder for this preprint (which was not certified by peer review) is the author/funder, who has granted bioRxiv a license to display the preprint in perpetuity. It is made available under aCC-BY 4.0 International license.

3	133.564	137.533	113.249	123.29	142.437
4	161.351	139.518	122.939	95.853	166.604
5	159.833	143.137	112.548	135.549	137.183
6	107.295	111.498	121.889	114.3	114.65
7	116.518	166.721	141.736	147.34	143.021
8	147.924	140.102	133.447	108.579	155.98
9	156.797	162.051	124.924	112.198	158.198
10	127.376	106.477	117.919	147.807	211.32
11	150.259	125.041	97.137	130.878	128.66
12	139.168	121.538	110.68	133.097	97.488
13	138.467	145.706	121.071	140.569	113.949
14	142.67	134.264	114.767	155.863	133.681
15	178.28	168.472	132.746	125.158	125.975
16	158.665	151.193	85.929	155.28	166.254
17	158.198	113.833	134.848	131.929	106.127
18	123.99	158.899	124.574	153.879	130.762
19	166.604	113.833	139.752	132.63	83.477
20	118.736	136.132	100.64	123.29	157.848
21	131.812	178.163	98.422	118.97	99.706
22	135.432	173.726	105.31	144.422	132.046
23	144.538	143.021	130.762	111.965	172.442
24	104.142	145.589	114.884	139.285	159.366

bioRxiv preprint doi: https://doi.org/10.1101/2024.02.14.580277; this version posted February 14, 2024. The copyright holder for this preprint (which was not certified by peer review) is the author/funder, who has granted bioRxiv a license to display the preprint in perpetuity. It is made available under aCC-BY 4.0 International license.

25	106.711	91.183	131.345	135.665	102.625
26	114.183	104.609	116.985	116.285	181.549
27	136.016	148.858	132.396	98.655	116.051
28	157.848	115.467	129.127	114.183	94.802
29	115.701	149.675	118.269	104.493	117.686
30	131.345	158.782	155.63	129.127	
31	92.817	146.757		142.904	
32	134.848	149.909			
33	114.65	127.026			
34	137.65	151.076			
35	184.818	149.442			
36		138			
37		134.965			
38		133.564			
39		185.401			
40		134.381			

nanos driven Set1 RNAi Data:

Testis #	Day 0	Day 1	Day 3	Day 5	Day 7
1	171.858	242.843	289.077	225.914	133.33
2	146.873	476.697	269.579	160.3	280.32
3	172.209	488.372	571.966	227.549	638.514

bioRxiv preprint doi: https://doi.org/10.1101/2024.02.14.580277; this version posted February 14, 2024. The copyright holder for this preprint (which was not certified by peer review) is the author/funder, who has granted bioRxiv a license to display the preprint in perpetuity. It is made available under aCC-BY 4.0 International license.

4	208.868	274.249	190.772	288.26	396.255
5	204.082	236.305	217.975	616.215	284.757
6	424.976	193.224	410.849	227.315	411.666
7	210.853	237.006	281.721	161.351	217.975
8	195.092	228.833	234.437	316.98	159.249
9	198.828	318.615	195.559	207.467	340.914
10	194.158	202.097	289.777	268.996	127.609
11	159.599	161.117	207.117	296.432	295.615
12	181.315	216.341	292.112	185.985	165.904
13	250.432	263.158	257.787	204.549	134.381
14	173.376	185.401	276.818	264.792	315.93
15	239.691	252.65	210.27	219.493	325.089
16	179.797	270.163	194.041	189.254	126.559
17	147.691	129.594	625.671	195.092	419.138
18	203.498	211.204	171.858	377.925	658.946
19	200.579	297.95	221.945	427.544	671.321
20	188.32	205.833	383.062	239.924	457.433
21	205.016	211.554	192.523	162.168	345.118
22	196.376	190.655	205.366	597.184	278.569
23	197.077	159.132	227.782	211.32	594.849
24	208.518	139.401	179.797	583.874	310.793
25	152.594	288.61	313.361	305.422	623.92

bioRxiv preprint doi: https://doi.org/10.1101/2024.02.14.580277; this version posted February 14, 2024. The copyright holder for this preprint (which was not certified by peer review) is the author/funder, who has granted bioRxiv a license to display the preprint in perpetuity. It is made available under aCC-BY 4.0 International license.

26	180.381	222.295	266.544	189.488	263.742
27	238.64	310.559	381.194	97.838	371.854
28	222.762	181.082	258.488	232.219	223.813
29	188.67	211.554	175.127	288.843	506.468
30	197.427	172.792	278.219	592.164	270.513
31	140.686	201.747	310.793	411.666	256.153
32	168.005	162.401	222.412	93.284	259.072
33	110.097	177.346	230.234	136.599	266.777
34	198.828	217.742		156.564	340.681
35	237.239			397.772	255.686
36	132.396			249.732	366.95
37	234.554			256.62	533.555
38	200.112			195.092	158.665
39	189.488			184.701	266.193
40	158.899			659.179	
41	242.61				
42	140.686				
43	186.452				
44	158.315				
45	109.63				
46	191.122				
47	175.478				

bioRxiv preprint doi: https://doi.org/10.1101/2024.02.14.580277; this version posted February 14, 2024. The copyright holder for this preprint (which was not certified by peer review) is the author/funder, who has granted bioRxiv a license to display the preprint in perpetuity. It is made available under aCC-BY 4.0 International license.

48	206.884				
49	186.102				
50	199.762				
51	143.254				

nos-gal4, tub-gal80^{ts} driven Control RNAi Data:

Testis #	Day 0	Day 7	Day 14	Day 21	Day 28
1	103.092	68.767	184.117	154.813	52.188
2	129.828	96.904	85.345	102.508	121.422
3	106.828	99.356	112.198	96.437	91.65
4	83.01	76.472	183.3	85.112	91.883
5	107.995	114.183	93.051	183.767	113.249
6	83.01	111.614	130.178	94.218	97.254
7	92	110.213	115.234	90.949	115.584
8	85.696	98.772	148.158	144.071	109.163
9	95.269	74.487	100.873	77.056	75.188
10	105.427	113.949	93.751	93.284	132.513
11	115.934	86.746	148.741	74.254	125.858
12	131.112	98.889	111.147	102.741	86.863
13	112.548	107.061	82.193	94.919	90.015
14	76.122	109.279	138.818	76.005	103.792
15	78.691	99.356	117.919	81.96	125.741

bioRxiv preprint doi: https://doi.org/10.1101/2024.02.14.580277; this version posted February 14, 2024. The copyright holder for this preprint (which was not certified by peer review) is the author/funder, who has granted bioRxiv a license to display the preprint in perpetuity. It is made available under aCC-BY 4.0 International license.

16	69.234	114.066	162.635	100.406	103.675
17	100.406	162.868	146.173	154.696	136.599
18	104.376	133.914	132.98	87.914	164.386
19	78.107	108.812	112.899	185.635	101.574
20	83.361	111.848	110.447	95.97	128.31
21	97.721	89.548	139.051	103.325	128.66
22	111.965	131.462	84.762	115.234	107.645
23	93.401	108.579	106.477	119.203	120.254
24	117.802	117.452	103.909	82.076	82.31
25	77.29	112.665	108.579	114.183	115.467
26	76.005	126.325	122.122	84.645	74.371
27	111.498	159.599	84.411	99.706	113.482
28	103.325	98.538	130.528	98.655	127.142
29	81.026	94.335	94.452	91.767	131.696
30	91.183	132.396	102.858	113.949	113.132
31		97.021	123.173		88.965
32					153.295

nos-gal4, tub-gal80^{ts} driven Set1 RNAi Data:

**Testis #**	**Day 0**	**Day 7**	**Day 14**	**Day 21**	**Day 28**
1	102.158	112.432	138.234	147.924	192.29
2	89.432	112.782	98.422	173.259	150.843

bioRxiv preprint doi: https://doi.org/10.1101/2024.02.14.580277; this version posted February 14, 2024. The copyright holder for this preprint (which was not certified by peer review) is the author/funder, who has granted bioRxiv a license to display the preprint in perpetuity. It is made available under aCC-BY 4.0 International license.

3	109.279	160.65	169.056	171.041	180.498
4	95.97	105.076	96.087	157.498	204.899
5	67.249	106.711	116.985	177.462	203.731
6	95.036	105.193	122.939	206.884	133.214
7	109.98	166.604	191.122	170.107	115.117
8	89.432	103.208	197.66	129.477	242.259
9	68.066	94.218	163.802	207.701	215.523
10	61.762	123.64	109.513	193.924	164.736
11	99.939	70.051	156.564	133.797	147.107
12	96.904	132.279	202.097	199.061	163.219
13	53.589	141.503	148.625	123.64	672.139
14	119.437	131.929	130.528	68.65	139.752
15	90.249	119.67	164.153	177.813	148.391
16	102.858	119.203	137.066	223.346	100.873
17	97.371	137.183	162.868	102.858	119.087
18	75.071	130.528	154.112	88.848	132.63
19	104.259	121.655	157.264	194.625	92.934
20	69.584	111.264	118.269	163.919	114.884
21	55.107	90.482	123.056	137.066	120.021
22	111.147	103.559	127.259	223.229	125.391
23	109.63		134.848	121.305	139.985
24	78.224		158.899	74.02	171.975

bioRxiv preprint doi: https://doi.org/10.1101/2024.02.14.580277; this version posted February 14, 2024. The copyright holder for this preprint (which was not certified by peer review) is the author/funder, who has granted bioRxiv a license to display the preprint in perpetuity. It is made available under aCC-BY 4.0 International license.

25	87.447		123.757	106.828	137.767
26	71.919		98.422	148.508	167.655
27	117.919		125.625	155.396	
28	109.279		229.3	112.782	
29	78.924		112.665		
30	84.995		158.549		
31	69		158.549		
32	53.355		205.366		
33	76.472		104.726		
34	95.736				
35	90.366				

tj driven RNAi Data:

Testis #	*Control RNAi*	*Set1 RNAi*
1	85.345	73.67
2	79.741	59.193
3	116.168	87.213
4	121.422	95.269
5	101.807	74.254
6	128.076	130.178
7	84.995	88.264
8	73.67	67.482

bioRxiv preprint doi: https://doi.org/10.1101/2024.02.14.580277; this version posted February 14, 2024. The copyright holder for this preprint (which was not certified by peer review) is the author/funder, who has granted bioRxiv a license to display the preprint in perpetuity. It is made available under aCC-BY 4.0 International license.

9	86.863	75.888
10	93.518	28.604
11	65.381	155.747
12	117.569	112.899
13	76.823	92.701
14	84.411	82.66
15	83.944	99.356
16	139.635	83.594
17	107.878	82.427
18	84.995	98.188
19	113.132	84.878
20	78.807	120.955
21	81.026	114.3
22	107.178	88.965
23	102.975	74.604
24	84.645	93.985
25	100.873	118.853
26	110.564	93.868
27	88.965	83.711
28	116.635	90.716
29	82.777	110.914
30	94.335	96.437

bioRxiv preprint doi: https://doi.org/10.1101/2024.02.14.580277; this version posted February 14, 2024. The copyright holder for this preprint (which was not certified by peer review) is the author/funder, who has granted bioRxiv a license to display the preprint in perpetuity. It is made available under aCC-BY 4.0 International license.

31	107.061	87.68
32	101.224	110.68
33	121.772	90.482
34	101.807	95.97
35	98.422	84.645
36	79.625	98.889
37	129.944	
38	86.863	
39	82.777	
40	81.493	
41	77.173	
42	89.081	
43	134.264	
44	109.513	
45	86.63	
46	74.954	

bam driven RNAi Data:

**Testis #**	***Control RNAi***	***Set1 RNAi***
1	119.67	92.35
2	127.259	120.721
3	87.213	144.305

bioRxiv preprint doi: https://doi.org/10.1101/2024.02.14.580277; this version posted February 14, 2024. The copyright holder for this preprint (which was not certified by peer review) is the author/funder, who has granted bioRxiv a license to display the preprint in perpetuity. It is made available under aCC-BY 4.0 International license.

4	123.173	107.178
5	142.904	162.635
6	121.772	141.386
7	117.452	113.366
8	141.62	106.244
9	115.818	83.711
10	99.589	100.29
11	167.772	126.442
12	125.274	113.482
13	118.736	105.894
14	81.96	78.457
15	115.584	146.873
16	128.076	87.914
17	155.747	110.33
18	84.411	135.549
19	110.33	126.442
20	118.386	138.351
21	127.259	126.909
22	95.503	137.533
23	104.609	107.295
24	98.889	128.894
25	198.478	88.264

bioRxiv preprint doi: https://doi.org/10.1101/2024.02.14.580277; this version posted February 14, 2024. The copyright holder for this preprint (which was not certified by peer review) is the author/funder, who has granted bioRxiv a license to display the preprint in perpetuity. It is made available under aCC-BY 4.0 International license.

26	163.919	131.579
27	134.848	101.807
28	100.29	114.884
29	98.538	99.589
30	129.944	96.67
31	113.015	98.538
32		140.919
33		83.361
34		114.3
35		150.96
36		148.158
37		118.736
38		137.533
39		114.65
40		167.772
41		103.792
42		129.244
43		168.472
44		135.198
45		83.477
46		128.66
47		102.041

bioRxiv preprint doi: https://doi.org/10.1101/2024.02.14.580277; this version posted February 14, 2024. The copyright holder for this preprint (which was not certified by peer review) is the author/funder, who has granted bioRxiv a license to display the preprint in perpetuity. It is made available under aCC-BY 4.0 International license.

48		120.488
49		93.985
50		115

nos-gal4, bam-gal80 driven RNAi Data:

**Testis #**	***Control RNAi***	***Set1 RNAi***
1	59.591	109.663
2	103.665	92.581
3	95.45	94.798
4	76.412	107.576
5	85.54	95.58
6	84.366	133.395
7	98.449	86.713
8	71.326	91.668
9	142.001	129.874
10	133.004	129.744
11	140.045	103.925
12	89.191	94.146
13	88.539	99.643
14	86.713	101.314
15	83.975	117.63
16	103.404	98.051

bioRxiv preprint doi: https://doi.org/10.1101/2024.02.14.580277; this version posted February 14, 2024. The copyright holder for this preprint (which was not certified by peer review) is the author/funder, who has granted bioRxiv a license to display the preprint in perpetuity. It is made available under aCC-BY 4.0 International license.

17	103.404	82.671
18	89.321	97.406
19	126.614	95.971
20	83.975	129.222
21	94.276	81.758
22	119.703	131.83
23	100.144	193.768
24	87.626	85.018
25	83.714	56.722
26	101.969	100.405
27	89.712	113.053
28	73.152	76.673
29	100.665	108.228
30	85.8	120.094
31	114.618	76.021
32	80.861	99.361
33	104.02	84.105
34	99.086	120.746
35	138.72	449.213
36	126.384	906.626
37	120.415	244.622
38	111.024	107.707

bioRxiv preprint doi: https://doi.org/10.1101/2024.02.14.580277; this version posted February 14, 2024. The copyright holder for this preprint (which was not certified by peer review) is the author/funder, who has granted bioRxiv a license to display the preprint in perpetuity. It is made available under aCC-BY 4.0 International license.

39	73.152	181.771
40	103.795	143.696
41	82.28	99.753
42	78.237	83.453
43	79.933	135.611
44	98.709	104.186
45	115.922	79.15
46	73.804	127.918
47	93.754	89.191
48	106.664	108.489
49	80.845	123.876
50	101.578	107.968
51	89.843	107.185
52	127.527	101.709

Table S3: Quantification of Cyst Cell number in RNAi knockdown testes.

nanos driven Control RNAi Data:

**Testis #**	**Day 0**	**Day 1**	**Day 3**	**Day 5**	**Day 7**
1	99	138	130	125	119
2	101	142	128	113	131
3	85	126	113	128	128
4	106	130	117	95	126

bioRxiv preprint doi: https://doi.org/10.1101/2024.02.14.580277; this version posted February 14, 2024. The copyright holder for this preprint (which was not certified by peer review) is the author/funder, who has granted bioRxiv a license to display the preprint in perpetuity. It is made available under aCC-BY 4.0 International license.

5	112	120	119	120	134
6	92	118	136	127	90
7	99	133	113	137	120
8	103	158	122	129	139
9	124	138	118	102	154
10	95	119	117	135	155
11	111	135	103	108	120
12	101	133	116	125	115
13	102	123	128	121	117
14	116	140	126	138	128
15	107	161	125	110	131
16	100	135	98	136	110
17	124	127	105	134	114
18	96	138	124	135	125
19	115	118	124	149	138
20	88	137	132	119	113
21	110	126	124	133	109
22	106	126	119	153	105
23	87	128	121	97	107
24	117	137	116	123	106
25	116	108	126	109	96
26	136	109	133	106	133

bioRxiv preprint doi: https://doi.org/10.1101/2024.02.14.580277; this version posted February 14, 2024. The copyright holder for this preprint (which was not certified by peer review) is the author/funder, who has granted bioRxiv a license to display the preprint in perpetuity. It is made available under aCC-BY 4.0 International license.

27	123	122	117	112	118
28	133	126	118	94	117
29	101	131	111	101	109
30	137	125	121	114	
31	78	120		101	
32	94	136			
33	113	111			
34	113	109			
35	140	95			
36		105			
37		109			
38		105			
39		156			
40		100			

nanos driven Set1 RNAi Data:

Testis #	Day 0	Day 1	Day 3	Day 5	Day 7
1	57	116	212	304	137
2	63	76	147	196	303
3	85	129	214	145	390
4	85	118	211	202	243
5	86	117	231	386	238

bioRxiv preprint doi: https://doi.org/10.1101/2024.02.14.580277; this version posted February 14, 2024. The copyright holder for this preprint (which was not certified by peer review) is the author/funder, who has granted bioRxiv a license to display the preprint in perpetuity. It is made available under aCC-BY 4.0 International license.

6	124	115	156	156	179
7	82	132	244	202	270
8	87	108	158	162	201
9	77	101	158	231	210
10	69	135	172	190	159
11	78	136	165	183	278
12	89	144	190	144	149
13	116	92	193	176	173
14	75	158	176	314	294
15	73	109	186	168	221
16	83	51	144	203	179
17	84	130	184	192	220
18	93	120	188	193	243
19	113	107	163	217	268
20	84	121	163	209	284
21	120	125	199	213	246
22	89	159	121	220	320
23	112	93	173	201	292
24	138	126	125	204	325
25	102	168	169	159	285
26	92	109	221	160	304
27	94	126	150	89	234

bioRxiv preprint doi: https://doi.org/10.1101/2024.02.14.580277; this version posted February 14, 2024. The copyright holder for this preprint (which was not certified by peer review) is the author/funder, who has granted bioRxiv a license to display the preprint in perpetuity. It is made available under aCC-BY 4.0 International license.

28	112	102	117	191	193
29	109	177	87	333	284
30	92	140	134	247	197
31	93	137	171	309	281
32	89	102	211	139	157
33	90	107	161	172	195
34	87	157		222	96
35	96			275	193
36	64			167	224
37	72			222	198
38	86			223	207
39	72			297	172
40	89			204	
41	90				
42	81				
43	83				
44	71				
45	81				
46	103				
47	71				
48	100				
49	65				

bioRxiv preprint doi: https://doi.org/10.1101/2024.02.14.580277; this version posted February 14, 2024. The copyright holder for this preprint (which was not certified by peer review) is the author/funder, who has granted bioRxiv a license to display the preprint in perpetuity. It is made available under aCC-BY 4.0 International license.

50	84				
51	91				

nos-gal4, tub-gal80^{ts} driven Control RNAi Data:

Testis #	Day 0	Day 7	Day 14	Day 21	Day 28
1	89	138	130	116	75
2	93	147	101	110	85
3	85	144	113	88	64
4	98	179	124	75	60
5	79	159	122	138	78
6	83	117	107	102	87
7	84	111	121	113	75
8	89	136	148	121	66
9	89	146	100	86	65
10	90	149	74	86	105
11	103	140	105	76	79
12	83	156	108	101	79
13	101	130	73	135	61
14	84	167	96	86	92
15	83	112	122	78	93
16	71	109	104	111	81
17	115	121	96	96	89

bioRxiv preprint doi: https://doi.org/10.1101/2024.02.14.580277; this version posted February 14, 2024. The copyright holder for this preprint (which was not certified by peer review) is the author/funder, who has granted bioRxiv a license to display the preprint in perpetuity. It is made available under aCC-BY 4.0 International license.

18	108	134	98	79	120
19	110	114	105	113	70
20	109	102	107	95	95
21	118	105	86	84	91
22	98	115	70	110	78
23	116	92	104	116	70
24	103	138	88	103	78
25	80	118	100	111	86
26	101	122	107	106	71
27	105	117	86	82	104
28	85	96	104	108	96
29	76	114	87	105	75
30	105	134	105	84	97
31		103	133		84
32					89

nos-gal4, tub-gal80^{ts} driven Set1 RNAi Data:

**Testis #**	**Day 0**	**Day 7**	**Day 14**	**Day 21**	**Day 28**
1	131	89	94	107	146
2	80	98	107	141	66
3	115	103	62	79	112
4	111	86	92	94	74

bioRxiv preprint doi: https://doi.org/10.1101/2024.02.14.580277; this version posted February 14, 2024. The copyright holder for this preprint (which was not certified by peer review) is the author/funder, who has granted bioRxiv a license to display the preprint in perpetuity. It is made available under aCC-BY 4.0 International license.

5	74	95	92	126	88
6	108	99	54	110	77
7	111	117	95	106	53
8	112	99	91	69	79
9	81	104	90	74	115
10	74	90	56	114	67
11	103	88	104	73	65
12	101	75	103	112	122
13	72	86	96	84	132
14	118	118	127	51	46
15	126	79	96	125	37
16	99	100	87	134	64
17	104	108	102	57	100
18	79	108	118	84	115
19	87	97	83	114	55
20	95	100	93	109	93
21	71	59	102	66	33
22	107	68	77	88	191
23	118		77	183	42
24	75		76	59	88
25	110		89	101	97
26	99		69	91	184

bioRxiv preprint doi: https://doi.org/10.1101/2024.02.14.580277; this version posted February 14, 2024. The copyright holder for this preprint (which was not certified by peer review) is the author/funder, who has granted bioRxiv a license to display the preprint in perpetuity. It is made available under aCC-BY 4.0 International license.

27	103		71	106	
28	108		82	109	
29	76		103		
30	92		67		
31	62		73		
32	79		109		
33	91		57		
34	82				
35	79				

tj driven Knockdown Data:

Testis #	*Control RNAi*	*Set1 RNAi*
1	88	86
2	78	72
3	85	103
4	68	66
5	57	84
6	77	73
7	91	83
8	81	95
9	85	75
10	90	45

bioRxiv preprint doi: https://doi.org/10.1101/2024.02.14.580277; this version posted February 14, 2024. The copyright holder for this preprint (which was not certified by peer review) is the author/funder, who has granted bioRxiv a license to display the preprint in perpetuity. It is made available under aCC-BY 4.0 International license.

11	69	107
12	77	105
13	61	98
14	65	114
15	82	98
16	78	88
17	102	115
18	99	97
19	84	94
20	67	101
21	68	127
22	96	97
23	97	96
24	96	104
25	83	101
26	100	133
27	86	111
28	93	132
29	69	123
30	96	103
31	79	
32	92	

bioRxiv preprint doi: https://doi.org/10.1101/2024.02.14.580277; this version posted February 14, 2024. The copyright holder for this preprint (which was not certified by peer review) is the author/funder, who has granted bioRxiv a license to display the preprint in perpetuity. It is made available under aCC-BY 4.0 International license.

33	127	

bam driven Knockdown Data:

**Testis #**	***Control RNAi***	***Set1 RNAi***
1	94	122
2	99	150
3	110	147
4	98	99
5	97	158
6	96	138
7	100	144
8	118	146
9	110	130
10	86	161
11	120	155
12	102	149
13	101	134
14	96	122
15	89	157
16	130	107
17	117	142
18	88	108

bioRxiv preprint doi: https://doi.org/10.1101/2024.02.14.580277; this version posted February 14, 2024. The copyright holder for this preprint (which was not certified by peer review) is the author/funder, who has granted bioRxiv a license to display the preprint in perpetuity. It is made available under aCC-BY 4.0 International license.

19	115	130
20	94	146
21	100	128
22	81	133
23	110	138
24	105	138
25	136	116
26	139	141
27	124	143
28	64	100
29	79	105
30	112	107
31	111	143
32		129
33		116
34		139
35		145
36		143
37		128
38		142
39		114
40		130

bioRxiv preprint doi: https://doi.org/10.1101/2024.02.14.580277; this version posted February 14, 2024. The copyright holder for this preprint (which was not certified by peer review) is the author/funder, who has granted bioRxiv a license to display the preprint in perpetuity. It is made available under aCC-BY 4.0 International license.

41		114
42		132
43		122
44		174
45		105
46		142
47		141
48		131
49		117
50		112

nos-gal4, bam-gal80 driven Knockdown Data:

**Testis #**	***Control RNAi***	***Set1 RNAi***
1	106	70
2	71	75
3	69	74
4	106	76
5	72	76
6	76	97
7	99	80
8	77	54
9	75	94

bioRxiv preprint doi: https://doi.org/10.1101/2024.02.14.580277; this version posted February 14, 2024. The copyright holder for this preprint (which was not certified by peer review) is the author/funder, who has granted bioRxiv a license to display the preprint in perpetuity. It is made available under aCC-BY 4.0 International license.

10	83	81
11	88	71
12	87	70
13	94	70
14	89	79
15	95	85
16	76	87
17	76	83
18	83	73
19	83	79
20	71	76
21	93	84
22	88	112
23	85	84
24	81	66
25	94	55
26	80	76
27	82	90
28	71	72
29	66	96
30	71	84
31	64	69

bioRxiv preprint doi: https://doi.org/10.1101/2024.02.14.580277; this version posted February 14, 2024. The copyright holder for this preprint (which was not certified by peer review) is the author/funder, who has granted bioRxiv a license to display the preprint in perpetuity. It is made available under aCC-BY 4.0 International license.

32	97	78
33	67	81
34	59	78
35	81	153
36	82	201
37	81	121
38	76	89
39	76	94
40	63	82
41	74	114
42	72	63
43	90	78
44	96	82
45	90	77
46	70	96
47	81	64
48	80	69
49	122	75
50	100	84
51	86	87
52	109	92
